# Magnetic Structure of Ion-Beam Imprinted Stripe Domains Determined by Neutron Scattering

**DOI:** 10.3390/nano10040752

**Published:** 2020-04-15

**Authors:** Thomas Saerbeck, Henning Huckfeldt, Boris P. Toperverg, Arno Ehresmann

**Affiliations:** 1 Institut Laue-Langevin, 71 Avenue des Martyrs, CS 20156, CEDEX 9, 38042 Grenoble, France; boris@ill.fr; 2 Institute of Physics and Center for Interdisciplinary Nanostructure Science and Technology (CINSaT), University of Kassel, Heinrich-Plett-Straße 40, D-34132 Kassel, Germany; henning.huckfeldt@gmail.com (H.H.); ehresmann@physik.uni-kassel.de (A.E.); 3 Petersburg Nuclear Physics Institute, National Research Center “Kurchatov Institute”, 188300 Gatchina, Saint Petersburg, Russia

**Keywords:** magnetic patterning, ion bombardment, magnetic domain, magnetic domain wall, magnetization vector, exchange bias, polarized neutron reflectometry, polarized neutron off-specular scattering

## Abstract

We present a detailed analysis of the in-plane magnetic vector configuration in head-to-head/tail-to-tail stripe domain patterns of nominal 5 μm width. The patterns have been created by He-ion bombardment induced magnetic patterning of a CoFe/IrMn_3_ exchange bias thin-film system. Quantitative information about the chemical and magnetic structure is obtained from polarized neutron reflectometry (PNR) and off-specular scattering (OSS). The technique provides information on the magnetic vector orientation and magnitude along the lateral coordinate of the sample, as well as the chemical and magnetic layer structure as a function of depth. Additional sensitivity to magnetic features is obtained through a neutron wave field resonance, which is fully accounted for in the presented analysis. The scattering reveals a domain width imbalance of 5.3 to 3.7 μm of virgin and bombarded stripes, respectively. Further, we report that the magnetization in the bombarded stripe significantly deviates from the head-to-head arrangement. A domain wall of 0.6 μm with homogeneous magnetization direction is found to separate the two neighboring domains. The results contain detailed information on length scales and magnetization vectors provided by PNR and OSS in absolute units. We illustrate the complementarity of the technique to microscopy techniques for obtaining a quantitative description of imprinted magnetic domain patterns and illustrate its applicability to different sample systems.

## 1. Introduction

Magnetic materials with engineered magnetic textures on micrometer length scales [1,2,3] have attracted attention due to their possible application in lab-on-a-chip devices [4,5,6,7], controlled self-organization [8,9], magnonics [10], controllable optically active surfaces [11] and biosensor applications [12]. One key limitation for lateral magnetic structures is the small feature size, leading to superparamagnetic fluctuations, and the lateral topography, which can limit accessibility by near contact techniques and provide an additional static potential landscape. Ion bombardment induced magnetic patterning (IBMP) has shown to provide reproducible magnetic patterns on micrometer and sub-micrometer length scales with negligible topographic height variations [12,13,14,15]. The technique is based on magnetic textures engineered by light-ion irradiation of thin-film exchange bias systems in oriented applied magnetic fields. The three-dimensional stray field landscape emerging from magnetic charges in the topographically flat underlying material provides the ability to guide and manipulate magnetic particles and molecules over distances of several millimeters [6,9,13,16,17,18]. 

Exchange bias, in its basic form, originates due to the exchange coupling at the interface between a ferromagnet (FM) and an antiferromagnet (AF). The effect is observed as a shift of the hysteresis loop along the applied field axis after setting the AF. Typically, this setting takes place by cooling the AF through its blocking temperature in an applied field strong enough to saturate the ferromagnet. A more general approach subjects the AF to thermal activation over a defined time-period, which may not exceed the Néel temperature of the AF [19]. In the case of light-ion bombardment, changes in the exchange bias direction and magnitude have been explained by hyper-thermal effects and microscopic changes in the defect density at the interface between the FM and AF and deeper in the AF [20,21,22]. For IBMP, a protective photoresist mask forming the pattern is applied prior to the bombardment, which generates the magnetic texture. The resulting magnetically patterned but structurally continuous film may present a route to circumvent the superparamagnetic limit, but direct exchange interactions between atomic spins and dipolar effects in the film plane can significantly alter the magnetic properties. Further, the bombardment process may lead to irreversible structural and magnetic changes, such as swelling and decay in exchange bias or magnetization of the FM [20,23], which can lead to instabilities in the magnetic potential landscape. 

In order to understand and design efficient patterns and magnetic field landscapes emerging from the surface, a detailed understanding of the magnetic properties deep in the sample, at the interfaces and above the surface is indispensable. Several experimental techniques provide complementary information on magnetization vectors and their lateral arrangement. While volume-averaging magnetometry techniques, such as superconducting quantum interference device (SQUID) measurements and vibrating sample magnetometry (VSM), can provide absolute values for the magnetization, magnetic texture characteristics can only be conjectured from the hysteresis curve determined by these methods. Microscopic characterization of the magnetization below the surface can be performed using magneto-optical Kerr microscopy (MOKE), x-ray photoemission electron microscopy (X-PEEM) [24,25] and scanning electron microscopy with polarization analysis (SEMPA) [26,27]. These provide a real-space image of the patterns, but provide no quantitative information about the magnetic vector lengths. Quantitative investigations above the surface of the sample are more involved, especially at defined distances and over large areas. Substantial efforts have been devoted to the quantitative measurement of the magnetic potential landscape provided by magnetic stray fields emerging from charged magnetic domain walls in different configurations. Magnetic force measurements with calibrated tips [28], magnetoresistive microscopy [14] and μ-Hall measurements [29] provide quantitative measures of the magnetostatic stray fields emerging from the surface with high spatial resolution. These measurements depend highly on their calibration [13] and only investigate the vacuum side of the film, which only allows indirect conclusions about the internal film structure. Further, the feedback between probe and surface must be taken into account. In general, microscopy and imaging techniques are surface sensitive techniques, which do not provide an extended resolution in depth of the sample. The element specificity of synchrotron-based X-PEEM provides some depth sensitivity, which is limited to the x-ray penetration and escape depth of the detected electrons. 

Specular polarized neutron reflectometry (PNR) and polarized off-specular scattering (OSS) provide quantitative information on the magnetic profile beneath the surface and therefore present a highly complementary technique for obtaining a three-dimensional picture of the magnetic pattern. The technique is non-destructive and measurements can be performed through different surrounding media, enabling in-situ application of different sample environments, for example liquid reservoirs for molecular self-assembly. PNR provides information on the laterally averaged magnetization of individual layers, depth resolved along the surface normal in a range from ~1 to ~300 nm total film thickness. OSS from lateral structures provides magnetization vectors, distances and in-plane correlations between domains or structural elements. While OSS can typically resolve structures between 500 and 100 μm, smaller structures can be investigated using grazing incidence neutron diffraction (GIND) [30]. The lateral and transverse resolution of PNR and OSS depends on the instrumental configuration, namely, the angular and wavelength resolution applied in the experiment. For a typical reflectometry setup, the resolution in depth amounts to 0.1 nm, while lateral length scales can be resolved with an accuracy of about 10 nm. 

In contrast to imaging techniques, PNR does not rely on relative contrast, but provides a measure of thickness and magnetization in absolute units related to the absolute contrast of the neutron scattering potential of the medium [31]. Quantitative information on the magnetization vector direction, lengths and distribution can be obtained from analysis of the scattered intensities as a function of external field and sample orientation [32,33]. The technique probes the full sample surface over centimeter lengths and therefore reveals the collaborative behavior of all domains in the sample. The applicability of the technique is not restricted to the materials discussed in this report, but comprises different systems in which a chemical or magnetic domain formation takes place on nanometer to micrometer length scales, as a function of depth or laterally. This includes applications in detection of lateral variations in exchange coupling, domain behavior in hard/soft magnetic exchange spring systems, correlated magnetization behavior in supported nanoparticle layers and magnetic domain distributions in emerging material systems, such as topological insulator or multiferroic thin film nanostructures. For recent reviews of the technique see [31,34,35,36,37,38,39] and references therein. 

**Figure 1:**
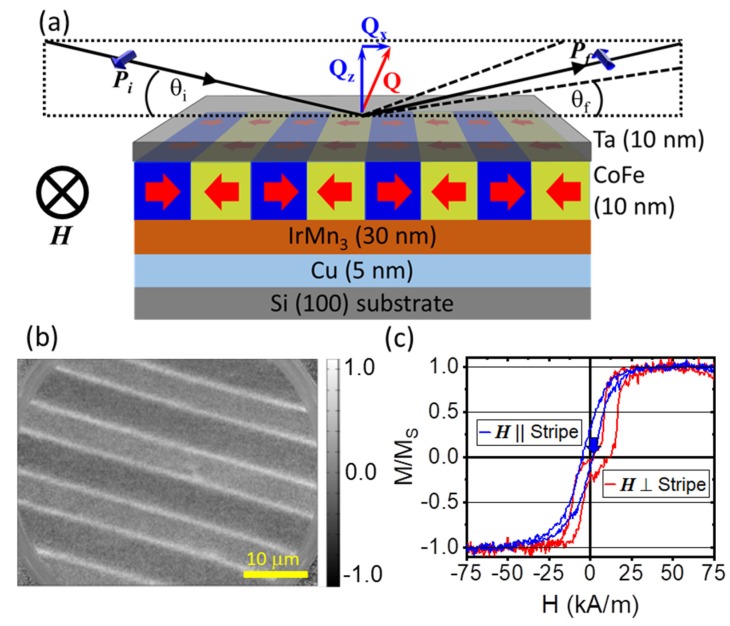
(**a**) Schematic sample structure and neutron scattering plane configuration perpendicular to the stripe axis. The magnetic field $H_{}$, which also defines the directions of neutron polarization vectors $P_{i}$ and $P_{f}$, is applied in the surface plane of the sample parallel to the stripe axis. (**b**) X-ray photoemission electron microscopy X-PEEM) image of the magnetic domain pattern and the domain walls separating two stripes. The color scale is normalized for the orientation between the photon wave vector surface projection $k_{\left|\right|}$ parallel to the long axis of the stripes. (**c**) Magneto-optical Kerr microscopy (MOKE) hysteresis with magnetic field applied perpendicular and parallel to the stripe axis, respectively.

Here we present a quantitative analysis of CoFe/IrMn_3_ magnetic stripe domains with nominal 5 μm width in a head-to-head/tail-to-tail (hth) configuration of magnetizations perpendicular to the long axis of the stripe (Figure 1a). Previous studies using neutron scattering on a similar system, albeit with magnetizations longitudinal to the stripe axis, revealed a complex reversal mechanism and a magnetization imbalance between the bombarded and virgin areas [32,33]. The samples do not present shape anisotropy, but individual domains can interact through direct exchange of atomic spins and dipolar interaction. In this respect, the present scenario presents a high-energy ground state and direct interactions between domains may influence the magnetic morphology. The co-refinement of the PNR and OSS simultaneously provides structural and magnetic information in absolute units as a function of depth and laterally along the sample surface. Further, we report on the observation of enhanced scattering due to a neutron wave resonance, which allows further quantitative insight into the magnetization distribution inside the sample. The analysis reveals stripes of (5.3 ± 0.1) μm and (3.7 ± 0.1) μm with magnetizations inclined 171° ± 4° to each other, separated by domain walls of substantial (0.6 ± 0.1) μm width. The magnetic moment within the stripe domains and the walls is of equal magnitude and only varies in orientation. With respect to the external field, the virgin domain is found to have an angle of 89° ± 3°, while the bombarded domain has a reduced canting of −82° ± 3°. The moment in the domain wall aligns parallel to the stripe axis. These orientations are homogeneous across the sample, leading us to the conclusion that deviations from the nominal state can be ascribed to small errors in the fabrication procedure rather than actual physical effects.

## 2. Materials and Methods 

The sample preparation followed the established protocol for IBMP of hth-domains described previously [14,23,29,40]. Exchange bias bilayers of nominally Ir_17_Mn_83_(30 nm)/Co_70_Fe_30_(10 nm) have been grown on Si (100) substrates with natural oxide, 5 nm Cu buffer, and 10 nm Ta capping layer. The initial exchange bias direction was set in two stages with application of an external field of 28 kA/m during deposition and field cooling in 70 kA/m from 300 °C with a magnetic field oriented parallel to the sample surface. Using UV lithography, stripe patterns with a nominal width of 5 μm were deposited. The orientation of the pattern was chosen such, that the long axis of the stripes runs perpendicular to the initial field cooling direction. The IBMP was performed with 10 keV He^+^ ions and a dose of 2 × 10^15^/cm^2^ in an applied field of 80 kA/m opposite to the initial field cooling direction and perpendicular to the stripe axis, which defines the hth domain configuration (Figure 1a). Finally, the resist mask was removed by subsequent sonication in 3% KOH, acetone and isopropanol. Following each step of the preparation process, VSM and atomic force microscopy (AFM) have been performed to monitor the magnetic behavior and confirm the intended surface structure of the resist pattern. The resulting sample surface was smooth with little amount of residual resist distributed over the sample surface as confirmed by AFM (not shown). 

The lateral magnetization profile was imaged using X-PEEM at the beamline UE56/1-SGM of the BESSY II synchrotron radiation facility (Berlin, Germany) [15,40,41]. The technique locally measures the partial electron yield (EY) after excitation with left or right circularly polarized x-rays of energies close to inner-shell absorption edges of the contributing atomic species. The magnetic contrast (I) defined as the asymmetry between the two respective helicities, is proportional the angle of the local magnetization and the surface projection of the photon wave vector $k_{\left|\right|}$, ($I\propto \cos\left(M\cdot k_{\left|\right|}\right)$ [24,41].

The volume averaged magnetic behavior was measured with VSM and vector magneto-optical Kerr effect (V-MOKE) [40,42]. Polarized neutron scattering studies were performed on the D17 beamline at the Institute Laue-Langevin in Grenoble, France [43]. The instrument operates with horizontal scattering geometry in time-of-flight mode (ToF) with a polarized wavelength band of 0.4–2 nm. A 2-dimensional detector with horizontal resolution of 2 mm FWHM at 3100 mm from the sample enables simultaneous detection of PNR and OSS signals [44]. The data was recorded at $\theta_{i}=0.5{}^{\circ}$ and $\theta_{i}=1.5{}^{\circ}$ angle of incidence in a magnetic field provided by an electromagnet at room temperature. The sample orientation was chosen such, that the long axis of the magnetic stripes is aligned with the external magnetic field and perpendicular to the scattering plane (Figure 1a). The incoming neutron polarization ($P_{i}$) was either parallel ($R^{+}$) or antiparallel ($R^{-}$) to the external field. For the measurement in saturation at 380 kA/m, a third angle of incidence of $\theta_{i}=5.0{}^{\circ}$ was added to extend the Q-range for a more reliable analysis of the structural and magnetic profile, in particular the roughness of the layers as a function of depth. For the measurement in low fields, the sample was first saturated in a field of negative 400 kA/m and the field subsequently increased to the measurement value of positive 2.2 kA/m. This state is marked with a blue arrow on the hysteresis with the external field applied parallel to the stripes in Figure 1c. During the experiments reported here, no spin-analysis of the scattering was applied. The specular data was extracted using the COSMOS software (COSMOS v. 3.3.13, Grenoble, France) [45] and corrected for the inefficiency of the polarization devices of the instrument using prior calibration. Analysis of the specular data took place using the GenX fitting software (GenX v. 2.4.10, Uppsala, Sweden) [46], the results of which were independently confirmed by a routine based on the super-iterative formalism developed by one of the authors [47]. The OSS is obtained directly from the 2-dimensional detector by integrating the vertical, loosely collimated, dimension [44]. The analysis of the scattering uses a custom simulation package for specular and off-specular intensities, which is based on the distorted-wave Born approximation (DWBA) [39,48,49,50]. 

## 3. Results

### 3.1. X-PEEM and Volume Magnetometry

An example X-PEEM measurement of the magnetic stripe pattern in shown in Figure 1b. The magnetic contrast in such measurements is determined by the orientation of the surface wave vector projection $k_{\left|\right|}$ to the magnetization, where a maximum contrast is achieved for parallel to antiparallel alignment [24,41]. For the measurement, the sample was oriented with $k_{\left|\right|}$ parallel to the stripes and therefore perpendicular to the domain magnetization. This reduces the contrast between the domains, but reveals the domain walls separating the two stripes, the latter being visible as bright lines in Figure 1b. Due to a small misalignment in the magnetization vectors in neighboring domains, a small contrast difference between the domains remains visible. A rotational scan of the sample, taking images at several orientations of $k_{\left|\right|}$ with respect to the magnetization revealed a canting of 6.5° ± 1° from the domain wall normal of one domain. The magnetization vector of the other domain aligns with the normal to the domain wall. In addition to the quantification of the magnetization direction, the image shows a difference in width between the two domains of about 1.5 μm.

The volume averaged magnetic hysteresis of the hth-stripe pattern recorded with MOKE is shown in Figure 1c. Although superimposed by an appreciable noise, the results agree with measurements performed with VSM [13,40]. With a field applied perpendicular to the stripe axis a double hysteresis loop separated by the sum of exchange bias values is observed. This separation leads to a close-to zero remanent magnetization indicating nearly opposite alignment of neighboring stripe domains of equal width. Differences in the domain magnetizations, lateral variations in exchange bias and global uncertainties in the orientation of the unidirectional anisotropy and stripe axes can lead to a finite remanent magnetization and asymmetric reversal of the hysteresis loop. Such a situation is observed in Figure 1c, where hysteresis and remanence remain observable at zero applied fields. This indicates that the two stripe domains do not behave equally, a behavior which has been observed before [28,29]. Additionally, a slight misalignment of the two exchange bias directions in the stripes with respect to each other and the stripe axis can lead to a preferential magnetization not perpendicular to the stripe border, which creates a residual magnetic component in low fields. Such a magnetic orientation misalignment with respect to the external field is also visible in measurements with the external field applied along the stripe axis (Figure 1c). This measurement geometry is expected to lead to zero coercive field values and zero exchange bias observed in the hysteresis. Indeed, no split double hysteresis is observed, but a symmetric ferromagnetic-like hysteresis with non-zero coercive field and apparent shift towards negative field remains. From the perpendicular hysteresis loop, the coercive field values and exchange bias values are determined to H_EB_(right) = (−10.1 ± 0.4) kA/m and H_EB_(left) = (9.8 ± 0.2) kA/m, while the coercive fields of the two branches are determined to H_C_(right) = (5.5 ± 0.2) kA/m and H_C_(left) = (2.4 ± 0.2) kA/m. The value for the loop shift agrees for both parts of the hysteresis loop, but the coercive field differs by a factor of two for the bombarded and virgin regions. The longitudinal measurement reveals a loop shift by H_EB,L_ = (1.6 ± 0.4) kA/m and coercive field of H_C,L_ = (3.1 ± 0.2) kA/m. 

### 3.2. Polarized Specular Reflectivity

**Figure 2:**
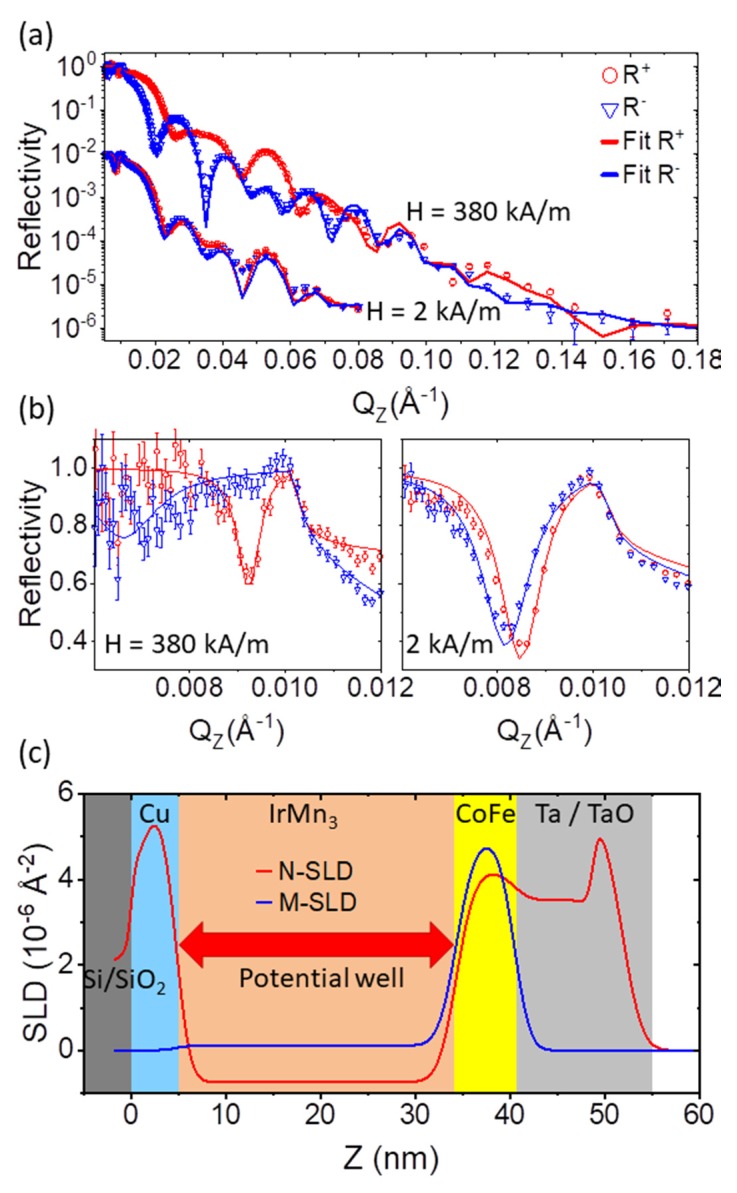
(**a**) Specular polarized reflectivity in saturation (380 kA/m) and near remanence (2 kA/m) (Circles:$\;R^{+}$; Triangles: $R^{-}$ ). The lines are fits to the data. (**b**) Enlarged view of the low $Q_{Z}$ total reflectivity region. (**c**) Scattering length density profile of the layer structure in saturation obtained from the fits to the data. The sample depth along the surface normal, with Z = 0 at the interface to the substrate, is displayed horizontally on the abscissa, while the SLD value is displayed on the ordinate. Shaded areas indicate the nominal layer sequence corresponding to the schematic shown in Figure 1.

Figure 2a,b show the specular reflectivity (SR) and fits to the data as a function of wave vector transfer $Q_{Z}=4\pi /\lambda \sin\theta$, with $\theta =\theta_{i}=\theta_{f}$, for two applied fields. Application of 380 kA/m in the sample plane saturates the magnetization along the applied field direction and removes the magnetic stripe domain texture. Then, no OSS is detected on the 2-dimensional detector. The sample consists therefore of a laterally homogeneous nuclear (N-SLD) and magnetic scattering length density (M-SLD), which can be approximated by a slab model. In contrast to x-ray reflectometry (XRR), whose scattering cross-section is determined by the electron density, the neutron cross section is determined by the bound coherent neutron scattering length, defining the nuclear scattering length density:

(1) $$N-\mathrm{SLD}=\frac{1}{V}\overset{n}{\underset{i}{\sum }}b_{i},$$

where V is a unit cell volume containing n atoms with neutron scattering length $b_{i}$. The value of b can vary strongly between neighboring elements in the periodic table, which makes PNR particularly more sensitive to interfaces of materials with similar electron densities than XRR. Similarly, the M-SLD can be described using the same unit cell volume,

(2) $$M-\mathrm{SLD}=\frac{1}{V}C\overset{n}{\underset{i}{\sum }}\mu_{i}=C^{\prime }m,$$

with $C=2.645\times 10^{-5}Å\mu_{B}^{-1}$ if the atomic magnetic moment $\mu_{i}$ is known in units of $\mu_{B}$, or $C^{\prime }=2.853\times 10^{-12}{Å}^{-2}A^{-1}m$ if the volume magnetization m is known in units of A/m [34,39]. This M-SLD is of similar magnitude to the N-SLD, making PNR particularly sensitive to magnetic structures. 

The SLD profiles corresponding to the best fit of the sample structure and its magnetization at saturation are shown in Figure 2c. The values obtained for thicknesses of the layers are close to the nominal values, with roughness values below 1.5 nm (Table 1). The modeling of the roughness follows the Névot–Croce approach, which takes into account an effective smearing of the SLD profile due to interface height variations or diffusion normal to the interface [51]. The width of the interfaces is modeled according to an error-function with a width $\sigma =\sqrt{\left\langle h^{2}\right\rangle }$, which corresponds to the root-mean square roughness. The approach assumes a Gaussian distribution of random fluctuations h of local interface positions around a laterally averaged mean position of the interface. The impact of this laterally averaged roughness is visualized in the density profile as a function of depth for all layers (Figure 2c). It is deduced from the least square fit of data in Figure 2a, where roughness mostly affects the behavior of the reflectivity curve at higher wave vector transfer $Q_{Z}$. In contrast, resolution mostly determines the sharpness of Kiessig fringe minima. A nonlinear least square standard routine has been applied to fit a theoretical PNR curve, calculated for the model SLD in Figure 2c and convoluted with the resolution function corresponding to the instrument configuration [44,45], to our data. The resolution function includes the divergence of the incoming beam, the detector resolution and the wavelength resolution. Hence, the sensitivity of the fit to the model parameter is expressed via the error bars ascribed to this parameter, while the fit quality is estimated by the $\chi^{2}$ normalized to the number of fit parameters. Roughness parameters where determined with the accuracy of about 10% which is mostly determined by relatively low statistics of data and number of points in the tail of the reflectivity curve, but not due to the finite resolution.

The surface of the Ta capping layer shows an increase in N-SLD due to a certain degree of oxidation with inhomogeneous stoichiometry as a function of depth. The literature value for full oxidation into Ta_2_O_5_, for example, provides a literature N-SLD of $4.85\times 10^{-6}$Å^−2^, which is at the lower limit of the determined error on the fit parameter. Such a larger variation from the nominal value could also arise from the post-growth treatment, including the lithography and etching processes, leading to a significant modification of the sample surface. A correlation between thickness, roughness and N-SLD values describing this surface layer, which is best represented with the profile shown in Figure 2c, prevents a more accurate determination of these parameters. The exact N-SLD of the surface layer or the correlation between its roughness and N-SLD does not influence the determination of the other layer parameters or conclusions presented in this report. Other SLD values are close to literature values for the elemental composition (Table 1) and within the range of values observed for untreated films of the same composition. Therefore, we assume that the sample treatment did not lead to drastic changes in the structural and chemical quality of the sample. In particular, we do not observe an enhanced diffusion region at the FM/AF interface, which was observed to increase significantly for doses above 1 × 10^15^/cm^2^ [20]. This indicates that the applied dose is still at the lower limit for enhanced intermixing at the CoFe/IrMn_3_ interface. Further indication for the absence of significant structural and magnetic modifications due to the He^+^ bombardment is obtained from the absence of OSS in the saturating external fields, discussed in more detail below. 

The splitting between $R^{+}$ and $R^{-}$ reflectivities, the spin-asymmetry, is indicative of the total magnetization of the layer structure and therefore proportional to the laterally averaged M-SLD of the slabs. The moment obtained from fitting the data amounts to (2.11 ± 0.05) μB/CoFe, which corresponds to a volume magnetization density of 1700 kA/m. In addition, the fit significantly improves if a small positive moment, corresponding to (0.3 ± 0.05) μ_B_/IrMn_3_, is attributed to the nominally AF layer. Similar to the N-SLD, we do not observe a drastic variation in the magnetic parameters due to the ion bombardment. This is largely in agreement with magnetometry studies, which after the same exposure of 2 × 10^15^ ions/cm^2^ observe only little variation in the saturation magnetization [23]. However, it should be noted that an investigation of ion-bombardment effects in the materials is not in the scope of this report and the effect of defect-density and magnetization variations have not been investigated systematically as a function of different He-ion exposure using PNR. By direct comparison of reflectivity profiles prior and post bombardment a variation of above 1% on lateral average in the N-SLD and M-SLD would be detectable by the technique. For the present study, the dose was intentionally kept at a lower level in order not to induce drastic changes in the film quality and avoid lateral chemical variations in addition to the lateral magnetic texture.

At $Q_{Z}$-values below the critical wave vector transfer of total reflection, $Q_{C}$, a non-flat reflectivity is observed (Figure 2b), which indicates a resonance structure within the SLD profile [53]. Such resonance features provide added sensitivity to certain aspects of the sample structure due to resonant enhancement of the neutron wave field in the sample at certain depth [54,55]. The potential well is created by the negative N-SLD of IrMn_3_ and the two positive boundaries formed by Cu on the one and CoFe on the other side as indicated in Figure 2c. The height of the right boundary is highly sensitive to the magnetization of CoFe. Because the magnetic potential is added or subtracted from the nuclear potential depending on the alignment of the neutron polarization $P_{i}$ and the magnetization, the location and shape of the resonance feature is different for the two incoming neutron polarizations. In addition to the enhanced sensitivity to the magnetic moment in the sample, the absorption cross-section of CoFe and IrMn_3_ becomes apparent. For the materials in the current sample, those are orders of magnitude smaller than the nuclear cross section and typically below the detection threshold in neutron reflectometry. Due to the resonance, the absorption cross section creates the dip in total reflection. In the fitting of the data, the absorption enters the model via the imaginary scattering length density (iN-SLD). Although the imaginary part has been allowed to vary independently from the nuclear part, fitting provides values close to literature values [52]. The fitting errors listed in Table 1 illustrate the sensitivity to the iN-SLD within the stack. Because the neutron absorption is wavelength dependent, the specular fits have been performed for each angle separately.

In reduced external magnetic field of 2.2 kA/m the splitting between the R^+^ and R^-^ reflectivity curves collapses (Figure 2a). As the reflectivity was recorded without polarization analysis, and therefore spin-flip processes are not resolved, the data do not allow concluding directly on domain formation or coherent rotation of the magnetization. However, with respect to the imprinted stripe domain patterns and the observed OSS discussed below, the domain formation is the likely scenario.

### 3.3. Coherence and Averaging in PNR

For the interpretation of the magnetic component of specular reflectivity lateral averaging inherent to SR and the resulting observables need to be discussed. The area of the averaging depends on the incoming neutron wave vector, namely its resolution in $\theta_{i}$, $\theta_{f}$, and λ. The neutron coherence volume projected on the sample surface takes the shape of an elongated ellipsoid with a length of about $l_{X}\sim 50$ μm in forward direction, but only few nm width perpendicular to the scattering plane ($l_{Y}$) [35,39,47,56]. In depth, the coherence length $l_{Z}$ extends over a fraction of a micrometer. Therefore, each coherence volume comprises only a small fraction of the sample volume illuminated by the incident beam. The measured SR and OSS intensities result from incoherent summation of intensities independently radiated from a large number of coherence spots covering the sample. 

Within each coherence volume, neutron waves interfere, providing a coherent enhancement of neutron flux scattered into directions of SR, refraction, diffuse OSS and Bragg diffraction, while suppressing scattering in all other directions in which interference is destructive. SR and refraction are due to the part of the optical potential independent of lateral coordinates. This part is described by the mean potential averaged over the coherence volume. By definition, the mean value is invariant with respect to its shift parallel to the surface, hence conserving the lateral projection of the wave vector ($Q_{X}=0$). Together with the conservation of energy, this results in Snell’s law of specular reflection. 

**Figure 3:**
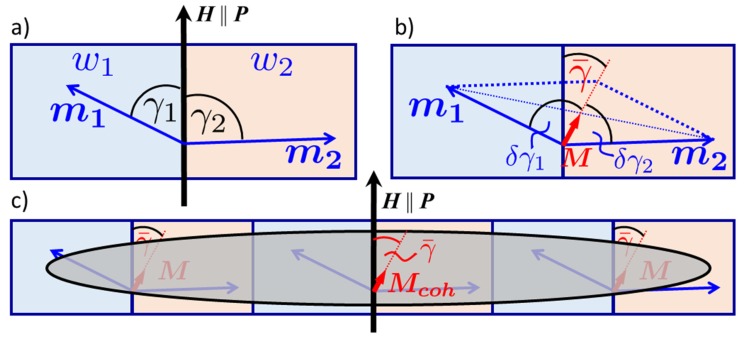
Example of coherent averaging in case of periodic arrays of magnetic domains. (**a**) Magnetic unit cell containing two domains of width $w_{1,2}$ with magnetization vectors $m_{1,2}$ (blue arrows), which are tilted by an angle $\gamma_{1,2}=\bar{\gamma }+\delta \gamma_{1,2}$ with respect to the external field H. (**b**) The angle $\bar{\gamma }$ is the angle of the average unit cell magnetization $M=\left(w_{1}/d\right)m_{1}+\left(w_{2}/d\right)m_{2}$ (red arrow), where $d=w_{1}+w_{2}$ is the period, and the external field H. Each domain magnetization $m_{1,2}$ is tilted by an angle $\delta \gamma_{1,2}$ with respect to this mean magnetization M. (**c**) Within the model for specular data, all magnetizations M within the coherence ellipsoid are averaged over the coherence area (grey shaded area). The coherence ellipsoid covers several magnetic unit cells and therefore could lead to a difference in $\bar{\gamma }$ with respect to (**b**) if not all unit cells magnetizations M are the same.

The magnetic part of mean optical potential of the coherence volume is proportional to its magnetization. The mean magnetization direction sets the quantization axis for the neutron spins and provides the splitting of the neutron spin states. This splitting is proportional to the absolute value of magnetization even though its direction may be tilted at an angle $\bar{\gamma }$ against the direction of external field guiding the neutron polarization. If the magnetization within the coherence volume is decomposed into a set of magnetic domains, the magnetization direction of each individual domain in the coherence volume may deviate from the direction of the mean magnetization by the angle δγ. The tilt angle of each domain magnetization vector against the external magnetic field can therefore be described by $\gamma =\bar{\gamma }+\delta \gamma$, where $\bar{\gamma }$ is the mean value of the tilt angle in a coherence volume averaged over deviations  δγ within (Figure 3). Such deviations δγ cause magnetic OSS, which can be diffuse if the domains are randomly distributed, or concentrated in Bragg diffraction peaks, if the domain magnetization varies periodically along surface. The latter case is illustrated in Figure 3a, where a schematic magnetic unit cell with two magnetizations $m_{1,2}$ at an angle $\gamma_{1,2}$ with respect to the external field is shown. The average of the two magnetizations leads to a drastically reduced mean magnetization M (Figure 3b), which itself makes an angle $\bar{\gamma }$ with the external field. The domain magnetizations can now be described by an angle $\delta \gamma_{1,2}$ with respect to M. Due to the extended length of the coherence ellipsoid, several magnetic unit cells are covered and contribute to the mean magnetization $M_{coh}$ (Figure 3c). 

The local variations in magnetization directions δγ within domains reduce the magnitude of the mean magnetization averaged over the coherence volume. The projection of domain magnetization onto the external field direction is proportional to the mean value of $\cos\gamma =\cos\bar{\gamma }\cos\left(\delta \gamma \right)-\sin\bar{\gamma }\sin\left(\delta \gamma \right).$ After averaging over the coherence volume the second term in this equation vanishes, because the equation $\bar{\sin\left(\delta \gamma \right)}=0$, where the bar indicates the coherent averaging over the coherence volume, actually determines the angle $\bar{\gamma }$. The factor $\bar{\cos\left(\delta \gamma \right)}\leq 1$ reduces the mean magnetization of the coherence volume and hence the splitting between spin states in the mean magnetic induction. Such a reduction manifests in a diminished spin splitting between critical wave vectors $Q_{c}{}^{\pm }$ of total reflection for the two incoming polarization directions. If the reduction factor $\bar{\cos\left(\delta \gamma \right)}\leq 1$ is unique for all coherence volumes then the parameter $\langle \cos\bar{\gamma }\rangle \neq 1$, where angular brackets denote incoherent averaging over the whole surface. The observed spin asymmetry, $\left(R^{++}-R^{--}\right)$, is directly proportional to $\langle \cos\bar{\gamma }\rangle$. 

For example, if the sample is totally demagnetized over distances greater than the coherence length in each direction and $\langle \cos\bar{\gamma }\rangle =0$, the reflectivities $R^{++}$ and $R^{--}$ merge into a single curve and the spin asymmetry vanishes. However, if at the same time $\bar{\cos\left(\delta \gamma \right)}\neq 0$, $R^{++}=R^{--}$ will still reveal two critical edges $Q_{c}{}^{\pm }$ and two plateaus of total reflection, at $Q\leq Q_{c}{}^{-}$, where $R^{++}=R^{--}=1$, and at $Q_{c}{}^{-}\leq Q\leq Q_{c}{}^{+}$. Due to the incoherent sum of intensities originating from the coherence volumes, both critical edges and plateaus can also be observed when $\langle \cos\bar{\gamma }\rangle \neq 0$. Only if $\langle \cos\bar{\gamma }\rangle =1$ the edge $Q_{c}{}^{+}$ is unique to $R^{++}\left(Q_{Z}\right)$, while $Q_{c}{}^{-}$ is revealed in that of $R^{--}\left(Q_{Z}\right)$. We should note that the condition $\langle \cos\bar{\gamma }\rangle =0$ does not necessarily mean that $\langle \sin\bar{\gamma }\rangle =\pm 1$. For example, if the magnetization in half of the surface area is tilted at an angle $\bar{\gamma }=0{}^{\circ}$, while in the other half $\bar{\gamma }=180{}^{\circ}$, then $\langle \cos\bar{\gamma }\rangle =0$ and $\langle \sin\bar{\gamma }\rangle =0$. Alternatively, if $\bar{\gamma }=\pm 90{}^{\circ}$, then $\langle \cos\bar{\gamma }\rangle =0$ and $R^{++}=R^{--}$, but one may expect $\langle \sin\bar{\gamma }\rangle =\pm 1$. This expectation can be proven by measuring spin-flip (SF) reflectivities $R^{+-}$ and $R^{-+}$ which are due to components of mean magnetization normal to the polarization vector. Due to symmetry reasons, SF reflectivities $R^{+-}=R^{-+}$ in one-dimensional polarization analysis [57] are equal to each other and proportional to $\langle \sin^{2}\bar{\gamma }\rangle$. Therefore, PNR with only one-dimensional analysis is not able to distinguish between right and left tilt of magnetization direction.

Without polarization analysis, the reflection into non-spin-flip channels (NSF) $R^{++}$ and $R^{--}$ and SF reflectivities $R^{+-}=R^{-+}$ contribute to the measured reflectivities $R^{+}=R^{++}+R^{+-}$ and $R^{-}=R^{--}+R^{-+}$. In such reduced version of PNR one can experimentally determine two statistical parameters: expectation values $\bar{\cos\left(\delta \gamma \right)}$ and $\left\langle \cos\bar{\gamma }\right\rangle$ characterizing the magnetization distribution over domains. $\bar{\cos\left(\delta \gamma \right)}$ is deduced from the splitting of critical edges of total reflection, $Q_{c}{}^{+}-Q_{c}{}^{-}$, of $R^{+}\left(Q\right)$ and $R^{-}\left(Q\right)$, while $\langle \cos\bar{\gamma }\rangle$ can be extracted from the spin asymmetry ($R^{+}-R^{-})$. However, the third statistical parameter, $\langle \sin^{2}\bar{\gamma }\rangle$, is only accessible with a complete version of one-dimensional PNR including polarization analysis. This parameter is important because it determines the dispersion $\langle \cos^{2}\bar{\gamma }\rangle =1-\langle \sin^{2}\bar{\gamma }\rangle$, which, along with the expectation value $\langle \cos\bar{\gamma }\rangle$, fully describes the distribution of magnetization projections onto the external field direction in case of Gaussian statistics.

Similarly to SR, one should distinguish between NSF and SF OSS originating from deviations of the magnetization directions in domains from that averaged over the coherence volume. A variation in magnitude of the domain magnetization projection onto the direction of the mean magnetization of the coherence volume contributes to NSF OSS. These variations do not mix spin states which are split in the mean magnetic induction. Components normal to the mean induction cause transitions between the two spin states. Therefore, if the mean induction in all coherence volumes is parallel to the external field, $\bar{\gamma }=0$, SF OSS is proportional to the mean value $\bar{\sin^{2}\left(\delta \gamma \right)}$, characterizing correlations of domain magnetization projections normal to the mean magnetization. Similarly, NSF OSS is due to correlations of longitudinal components, whose statistics is characterized by the mean value $\bar{\cos^{2}\left(\delta \gamma \right)}\;$ − $\;{\bar{\cos\left(\delta \gamma \right)}}^{\;2}$. The value $\bar{\cos\left(\delta \gamma \right)}$ is determined from the fit of specular PNR. Due to the constrain $\bar{\sin^{2}\left(\delta \gamma \right)}=1-\bar{\cos^{2}\left(\delta \gamma \right)}$, NSF OSS is actually described by the same statistical parameter $\bar{\sin^{2}\left(\delta \gamma \right)}$ as SF OSS. This parameter, as we will discuss in Section 3.4, can be extracted from the fit of OSS data collected in the reduced version of PNR without analysis of scattered neutrons.

Other parameters determining OSS are related to the distribution of magnetization over the ensemble of domains within the coherence ellipsoid. In the simplest case of random distribution, OSS is diffuse, with a line shape determined by the mean domain form-factor. The latter is described with one parameter corresponding to the inverse value of the mean size of domains. In general, the cross section of OSS is proportional to a linear combination of transverse and longitudinal correlators. The correlator for SF OSS, $G_{⊥}\left(Q\right)=\bar{m_{Q}^{⊥}m_{-Q}^{⊥}}$, describes correlations of the Fourier components of magnetization projections $m^{⊥}\left(r\right)=\left|m\right|\sin\left(\delta \gamma_{r}\right)$ onto the normal to mean magnetization. Similarly, the NSF OSS cross section is proportional to the correlator $G_{\left|\right|}\left(Q\right)=\bar{m_{Q}^{\left|\right|}m_{-Q}^{\left|\right|}}-M^{2}$ of deviations in longitudinal components $m^{\left|\right|}\left(r\right)=\left|m\right|\cos\left(\delta \gamma_{r}\right)$ of the domain magnetization vector m(r) from its mean value M. If components of m(r) are periodic functions of the distance r between domains then OSS cross section reveals a series of Bragg peaks of different orders. Their relative intensities are determined by the unit cell form-factor and the structure factor. Simultaneously, they are firmly scaled to the peak intensity of SR, which is the Bragg peak of the zeroth order. The latter is calculated exactly, while higher orders are described within the framework of DWBA.

The fitting of the SR data in saturation shown in Figure 2a,b provided a set of structural parameters collected in the Table 1, which has subsequently been fixed in fitting of the SR in low field. The fitting of the data obtained at 2.2 kA/m provided $\bar{\cos\delta \gamma }=0.160\pm 0.004$, which reveals a dramatic reduction of the mean value of the M-SLD averaged over the coherence volume and corresponds to a residual splitting of 0.34 μ_B_/CoFe. Additionally, the fitting provided $\langle \cos\bar{\gamma }\rangle =0.995\pm 0.003$, which indicates deviations of the mean magnetization direction amount to $\bar{\gamma }\approx \pm \left(5.7{}^{\circ}\pm 1.5{}^{\circ}\right)$. This means that the sample is mostly demagnetized within the coherence ellipsoids, while after averaging over all coherence areas only a small deviation of the mean magnetization from the external field direction remains. We note that the parameter $\langle \cos\bar{\gamma }\rangle$ correlates with the incident beam polarization and therefore depends on the precision of the correction for the inefficiency of the devices on the instrument. The values obtained from the fit of SR where used in the analysis of the OSS presented in Section 3.4. 

**Figure 4:**
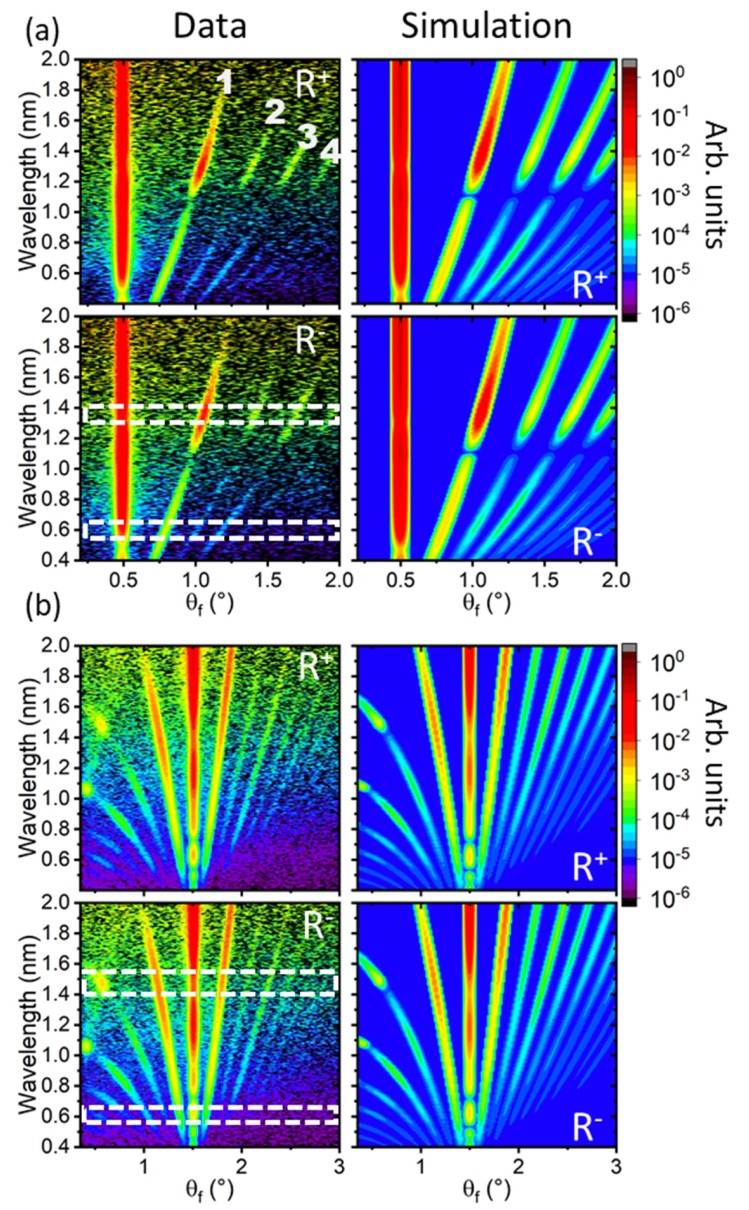
Contour plots of the off-specular intensity data observed at 2 kA/m applied external field (left column) and simulation (right column) at the two angles of incidence $\theta_{i}=0.5{}^{\circ}$ (**a**) and $\theta_{i}=1.5{}^{\circ}$ (**b**) in the two spin channels $R^{+}$ and $R^{-}$. The first four orders of Bragg diffraction are labelled in (**a**). The dashed boxes across the $R^{-}$ channels indicate the region of integration used for the line cuts in Figure 5. The color scale shows the scattered intensity normalized by the spectrum of the neutron beam.

Due to the reduced M-SLD, the potential well structure for “+” and “−” polarizations is altered with respect to that in saturation. The decrease in splitting indicates a multi-domain state in which the magnetization directions δγ deviate strongly from the external field direction, such that the mean magnetization averaged over several domains is close to zero. In the low field configuration, no magnetic moment in the IrMn_3_ was resolved by SR. In addition, the lateral domain structure creates scattering into the OSS channels (Figure 4), which is enhanced at the location of the resonance (Figure 2b). Next to the collapse in spin asymmetry, the reflectivity at the position of the resonance drops below 50%. The position of the dips in $R^{+}$ and $R^{-}$ are reproduced well with the reduced magnetic moment in CoFe, but the depth cannot be reproduced considering the nominal absorption alone. Instead, the scattering from the lateral structure needs to be taken into account to explain this feature. For an approximation of the loss in SR intensity due to scattering from lateral structures, an artificially enhanced effective absorption was introduced as discussed in more detail below. From the specular data alone, one cannot deduce the microscopic arrangement of magnetic states in the sample plane, which is responsible for the reduction in magnetization. 

### 3.4. Off-Specular Scattering

In saturating external magnetic fields (not shown), no off-specular intensity is observed even with prolonged acquisition times of 30 min compared to the 5 min used in measurements discussed below. This is in agreement with the AFM data and further shows that the observed residual resist patches on the surface do not contribute to the scattering. Further, this confirms that the individual layer roughness is uncorrelated in depth and laterally, and therefore does not lead to enhanced diffuse OSS intensities. The diffuse scattering arising from the individual layer roughness remains below the detection level as it is typically observed for non-correlated roughness. Furthermore, we do not observe any OSS from the topographic swelling of the stripe domains after IBMP or a notable decrease of saturation magnetization in bombarded regions [23]. Upon reduction of the external magnetic field, the magnetic stripe domains evolve at constant width and create the OSS pattern observed in Figure 4. The intensity maps shown are those also used to extract the specular reflectivity in Figure 2. For the quantitative analysis of the OSS intensities on the detector we remain in the instrumental $\left[\theta_{f},\lambda \right]$ coordinates which allows an enlarged view on the low-Q region near the total reflection [58]. A non-linear transformation into reciprocal space with $Q_{X}/Q_{Z}$ coordinates can be performed using the relationship

(3) $$Q_{Z}=\frac{2\pi }{\lambda }\left(\sin\theta_{i}+\sin\theta_{f}\right),\;\mathrm{and}$$

(4) $$Q_{X}=\frac{2\pi }{\lambda }\left(\cos\theta_{f}-\cos\theta_{i}\right).$$

This transformation changes the data point spacing for different wavelength and leads to a larger amount of triangulation for different Q-regions. In addition, the resolution function of $\theta_{i}$, $\theta_{f}$, and λ becomes a more complex function. In the instrumental coordinates, the data is contained in a linear matrix with constant pixel size for the full scattering region and the resolution function is known due to the divergence of the incoming beam and detector resolution [44,45]. In $\left[\theta_{f},\lambda \right]$ coordinates, the OSS is visible as curved lines around the specular reflectivity line located at $\theta_{i}=\theta_{f}$. Since no structural or chemical lateral contrast exists, the intensity of the Bragg diffraction lines depends on the magnetic contrast between domains given by the arrangement of magnetization vectors. At $\theta_{i}=0.5{}^{\circ}$ only the OSS towards larger $\theta_{f}$ is visible due to the horizon of the sample at $\theta_{i}=0{}^{\circ}$ being close to the specular reflection. In $Q_{X}/Q_{Z}$ coordinates the scattering would be visible as vertical lines with constant $Q_{X}\neq 0$ from which the period of the lateral elements can be determined with:

(5) $$Q_{X}=\frac{2\pi }{D}.$$

**Figure 5:**
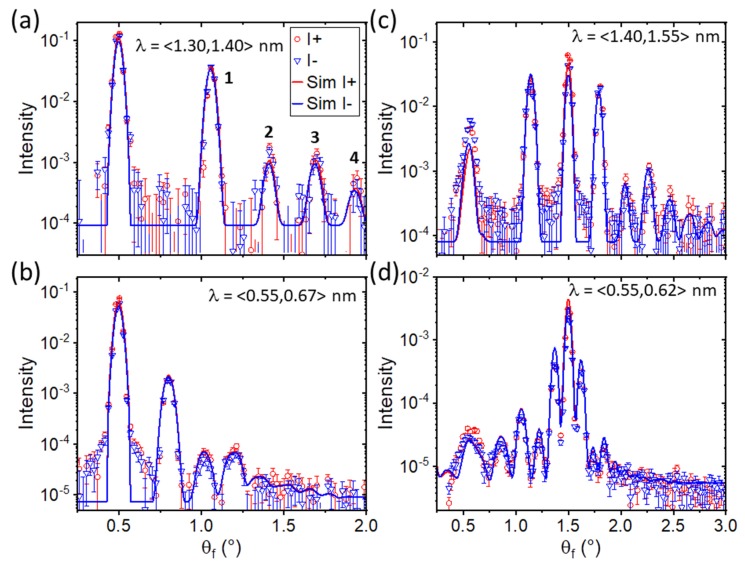
λ-cuts through the 2D data at 2 kA/m of Figure 4 integrating different wavelength regions indicated in the panels at an incident angle of 0.5° (**a**,**b**) and 1.5° (**c**,**d**). The order of the Bragg diffraction peaks is labelled in (**a**) according to Figure 4.

This provides an average period of *D* = (10.2 ± 0.2) μm for the repeating unit cell of two stripes. The contour plots have been normalized in wavelength by the direct beam spectrum [44] and only the instrumental background is subtracted, not the scattering from the sample or the sample holder. This background is constant in wavelength, but due to the normalization with the neutron spectrum appears to drop in intensity towards shorter *λ*. The simulations only include a constant background, whose value was adjusted for the different regions investigated in detail in Figure 5. 

Figure 4a shows data at an angle of incidence of $\theta_{i}=0.5{}^{\circ}$, which mainly comprises the total reflection plateau for the wavelength band shown. Typically, OSS is not observed below the critical wave vector transfer of total reflection $Q_{C}$, since the reflection amplitude is unity. Nevertheless, strong OSS is observed, which is a manifestation of the finite penetration depth of the neutron evanescent wave, whose scattering is enhanced by the resonance structure of the potential well. This is particularly visible around wavelength of 1.3 nm, which corresponds to the location of the dip in total reflectivity in reciprocal space (Figure 2). Above $Q_{Z}=Q_{C}$ the neutron wave fully penetrates the whole sample and the OSS intensity mainly follows the intensity profile determined by the cross section of the domain structure without the wave field enhancement (Figure 4b). The discrete nature of the intensity profiles, in contrast to diffuse scattering spanning a range of $\theta_{f}$, indicate a well ordered in-plane domain structure with defined width of each domain repeated laterally over large distances. In fact, the lines correspond to different orders of in-plane Bragg diffraction from periodic elements. In total, up to the 8th order diffraction can be identified at different angles of incidence, labelled up to the 4th-order in Figure 4a. The intense features at about 0.5°/1.5 nm and 0.4°/1.05 nm in Figure 4b are enhanced scattering due to the Yoneda effect [59], which results from the enhancement of the neutron wave field near $Q_{C}$ due to constructive interference between incident and scattered neutron waves. 

Even order Bragg diffraction is strongly suppressed by the structure factor of a periodically repeated unit cell containing two stripes of equal widths with opposing magnetization. The observation of strong 2nd-order and further even-order diffraction lines is therefore an indication of an imbalance between the domains either due to their magnetization or due to different widths. This imbalance can also create the residual splitting observed in the specular reflectivity. For the experiment, the external magnetic field was applied along the stripe axis and therefore perpendicular to the expected domain magnetization. This could lead to a different canting of magnetizations in the treated and virgin regions if the exchange anisotropy is not equal. In order to provide a quantitative measure for the scattered intensity constant wavelength cuts were made at several important regions of the $\left[\theta_{f},\lambda \right]$ contour plot. Figure 5a,b shows the intensity profiles obtained at $\theta_{i}=0.5{}^{\circ}$, while those obtained for the second angle of incidence, $\theta_{i}=1.5{}^{\circ}$, are shown in Figure 5c,d. Due to the integration, absolute normalization of the intensity with respect to the specular reflectivity is lost. The data is shown for both incoming spin states. Each integration was made over an interval of wavelengths as indicated in the figure to increase the statistics. The regions were chosen to comprise areas of strong OSS scattering and those, which are affected by wave field enhancements below and above $Q_{C}$ as indicated by the dashed boxes in Figure 4. Next to the specular intensity at $\theta_{f}=\theta_{i}$, Figure 5 shows strong off-specular Bragg diffraction including 2nd- and 4th-order peaks of similar intensity as the following odd-order diffraction peak. The scattering is similar in intensity for both incoming neutron spin polarizations. 

For the quantitative analysis of the polarized OSS, simulations based on the DWBA have been performed. Within DWBA, variations of the optical potential are considered as small perturbations to the mean optical potential of the sample. The analysis proceeds by first calculating the exact wave functions of the neutron propagating in the mean optical potential. The wave functions accounting for refraction through and reflection from laterally flat interfaces are further used as an input for the calculation of the cross section for OSS from the difference between the true profile of the scattering potential and its mean value averaged over the coherence ellipsoid. The difference is taken into account within a first order perturbation theory [39,47,48]. This means that the OSS is bound to the specular reflectivity, which is used as input for the laterally averaged potential. Similar to the slab model for specular reflectivity, a model system of lateral elements defines the potential landscape encountered by the neutron wave. These take the form of correlators of domain magnetizations described in Section 3.3, which are integrated in the simulation package designed by one of the authors. The simulation routine includes the resolution of the instrument in wavelength and angle, hence taking into account coherence properties of the neutron beam and providing an absolute scaling normalization of OSS to SR. 

Typically, OSS is by orders of magnitude lower in intensity compared to SR and transmitted intensities, which justifies the DWBA. A drawback becomes apparent in the current scenario where strong OSS arising from resonance structures in the sample leads to significant scattering away from the specular reflection (compare Figure 2 and Figure 5). Ignoring this effect in SR fitting violates the total flux conservation [60]. Such violation is only visible in the specular intensity in Figure 2b through the increased depth of the resonance dip in SR at low field. However, this effect is readily described by releasing the imaginary part of the SLD in the fit at low fields, which allocated an iN-SLD twice higher than nominally found to the IrMn_3_ layer. Thus, additional absorption compensates for the unaccounted losses in the specular intensity due to OSS substantially enhanced at resonance conditions. The specular fitting routine was free to allocate the additional absorption to all layers in the structure in order not to limit the generality of the approach. Further, the enhanced iN-SLD value found for IrMn_3_ does not influence other parameters determined from the SR fit. The validity of the approach to describe the scattering losses is confirmed by fitting specular reflectivities recorded at different external field values and different sample structures, which will be reported elsewhere [61]. A more rigorous approach accounting for the sum-rule of SR, transmission and OSS, required by the flux conservation law [60,62], can be accomplished in the second-order DWBA, which provides a non-zero imaginary part of the OSS amplitude even for a purely real scattering potential. In turn, the imaginary part of the amplitude in the forward direction is expressed in the total OSS cross section via the famous Optical Theorem (Bohr–Peierls–Placzek relation), hence formalizing a relationship between OSS into a solid angle and phenomenologically introduced absorption. The implementation of the second-order DWBA is beyond the scope of this report, but shall be highlighted here as one of the future tasks for the analysis of strong OSS from lateral structures.

**Figure 6:**
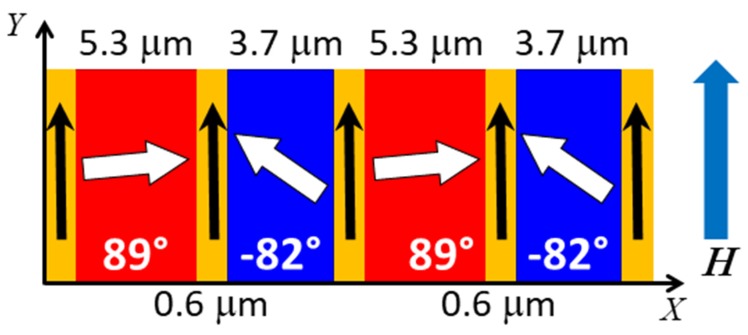
Schematic top view of the lateral domain configuration at 2 kA/m corresponding to the simulations in Figure 4 and Figure 5. X is defined as the lateral sample coordinate along the neutron beam projection onto the sample surface, while Y defines the direction perpendicular to the scattering plane (compare Figure 1). The external field H is applied along the stripe direction. The drawing is not to scale.

In the simulations of the OSS, the lateral potential was approximated with four purely magnetic elements repeated periodically over the sample surface. Note that structural elements, described by a lateral variation in N-SLD would lead to OSS also in saturation, which is not observed. Furthermore, the absolute magnetization of each CoFe domain was fixed to the value determined from specular reflectivity at saturation. The position of all Bragg peaks was reproduced with a period of the unit cell structure of 10.2 μm, which is in agreement with the value determined directly from the contour plots. The simulations proceeded by adjusting the domain width independently in order to create the even-order intensity. The intensity of the Bragg peaks and the residual magnetization observed in specular reflectometry are a result of individual tilt angles of magnetizations within domains, which define the scattering contrast. Interestingly, a variation of only the CoFe magnetization direction in two domains was found to be insufficient to match all observed peaks. The best agreement to the data was obtained with a four-element unit cell schematically shown in Figure 6, which is laterally repeated over the whole sample length. The model includes two domains of unequal widths, one being close to the nominal width of 5 μm, the other being substantially reduced to 3.7 μm. The magnetization direction in the large domain is tilted by an angle of $\delta \gamma_{1}=89{}^{\circ}\pm 3{}^{\circ}$ with respect to the net magnetization, which itself is tilted at an angle of $\bar{\gamma }\approx 5.7{}^{\circ}\pm 1.5{}^{\circ}$ against the external field and stripe axis (see Section 3.3). The magnetization in the smaller domain shows a tilt angle of $\delta \gamma_{2}=-82{}^{\circ}\pm 3{}^{\circ}$ in the opposite direction with respect to the same axis.

In addition, the model had to include intermediate regions between the domains, which can be interpreted as domain walls, of 0.6 μm width. The magnetization in this region is aligned with the external magnetic field and of the same magnitude as the domains. The projections of the mean magnetization deviations averaged over the domains, $\cos\left(\delta \gamma_{1}\right)=0.017$ and $\cos\left(\delta \gamma_{2}\right)=0.14$, and the two walls, $\cos\left(\delta \gamma_{W}\right)=1$, of the unit cell is consistent with the reduction factor $\bar{\cos\left(\delta \gamma \right)}=0.16$ for mean magnetic SLD deduced from the SR fit reported in Section 3.3. This reduction factor for the M-SLD explains the low, but still noticeable spin asymmetry in Figure 2 and Figure 5.

The sensitivity to the domain walls mostly arises from the resonance features located below $\theta_{f}=0.5{}^{\circ}$ in Figure 5c,d. These features are substantially broader and react strongly to the simulated width of the domain wall, the asymmetry between the domain widths and the tilt of the magnetization. A small improvement of the simulation could be observed by using the same moment of IrMn_3_ as determined in saturation, but with opposite sign in relation to the CoFe layer. However, with respect to the total match between simulation and data, this is not very reliable as the widths of the magnetic elements in CoFe and the canting angles have a much larger impact. The validity and sensitivity towards this small magnetization in IrMn_3_ is subject of a future study. We shall note that the sensitivity of PNR and OSS to the elements described in the model is not biased by findings from complementary techniques. In fact, the model has been gradually refined starting from a simple balanced two domain configuration in order to account for all features observed in Figure 2 and Figure 4. 

## 4. Discussion

The results show that we have succeeded in establishing a topographically flat array of stripes, with defined width, having a purely magnetic modulation. PNR determines a saturation magnetization of 1700 kA/m of the CoFe layer, which is the laterally averaged magnetization of the layer at any depth. This value is in agreement with the volume magnetization determined by VSM considering the thickness of the CoFe layer determined by PNR. The value provided by the fitted model (Figure 2c) is an absolute measurement and does not rely on exact volume normalization, as for example necessary for VSM measurements. Overall, reported VSM measurements show a substantial spread of values between 1200 kA/m [23] and 1800 kA/m [13], which can be related to uncertainties in the absolute magnetic layer thickness and the film quality. 

The PNR fit provides an N-SLD value which is slightly above the expected value for a Co_70_Fe_30_ composition and could therefore indicate a larger Fe concentration by about 3%, assuming a cubic unit cell with a lattice constant of 2.84 Å. However, it should be noted that PNR is not sensitive to elemental composition, but measures the average scattering length of elements in a given volume according to Equation (1). Therefore, the PNR measurement alone cannot distinguish between variations in scattering length or a change in density of atomic species in the unit cell. Scattering length and density contributions to the SLD can in principle be distinguished by co-refinement of XRR data using different energies due to the difference in scattering length of the probes. The absolute density and composition of the magnetic layer has no consequence on the conclusions made and therefore this absolute determination is out-of-scope for this report. The potential deviation from the nominal composition originates in the growth process and is laterally homogeneous in the sample and unrelated to the He-ion bombardment process. Also, we do not observe a change in the magnetic moment after the ion-bombardment, which indicates that the ferromagnetic layer remains close to the original composition during the patterning process. This conclusion is supported by the fact that no Bragg diffraction is detected with the sample in a saturated state. Such diffraction would be visible in case of different SLD values, either nuclear or magnetic, between bombarded and virgin neighboring stripes [63]. 

The magnetic vectors are oriented close to hth configuration, although deviations in one domain by 8° are observed. In addition, this domain also shows a significantly reduced width of 3.7 μm instead of the expected 5 μm as observed for the other domain. These values agree well with estimates made from X-PEEM images based on the grey-scale contrast, but provide absolute values of the domain canting angles and magnetization vector length. Further, the neutron measurements show that the saturation magnetization of the CoFe layer remains homogeneous across the whole sample and each domain contains the same magnetization vector length, but under a different orientation. 

The deviations in width of the domains are due to uncertainties in the fabrication procedure of the parallel-stripe domain texture: the parallel-stripe resist mask has been fabricated with a nominal thickness of about 700 nm in order to prevent the He-ions from penetrating into the magnetic layer system. Stripes of nominal width of 5 µm have been chemically etched into the resist, usually resulting in not absolutely steep mask walls. An inclination of only 5° already leads to an added footprint on one side of the stripe of about 60 nm. Moreover, the resist material typically charges upon ion bombardment, leading to reduced widths of the bombarded stripes due to Coulomb repulsion of the impinging ions on the surface. The deviations of the magnetic vectors can be explained by a misalignment of the fields used to initialize the exchange bias and the one used in the ion bombardment procedure. This misalignment leads to a residual magnetic moment of the sample in low external fields along a defined axis. The conclusion can be made that the domain of reduced width and canting angle is the one treated by ion bombardment as the resulting anisotropy can be weaker as in the virgin domain [20,23]. In general, the mechanisms of IBMP include local hyperthermal effects and defect creation, both of which can lead to a reduction of exchange bias depending on their location in the AF, FM or at the interface. Further, a small misalignment in the field direction during the IBMP may lead to a preferential orientation of the induced exchange anisotropy, which leads to an overall residual magnetic anisotropy component along the stripe axis.

Next to a reduced width of only one domain, smaller regions with different alignment of magnetizations could be identified, which we identify as domain walls. For thin film systems, a Néel wall is expected, which shows a rotation of the magnetization in the film plane [29]. The established width of 0.6 μm of the wall agrees with estimates made by MFM measurements of the stray field above the sample surface [13,14,15,28]. However, while these measurements only allow an indirect conclusion about domain walls, PNR and OSS probe the whole sample and provide a direct measure of the wall width, its magnetic vector length and orientation. The observed resonances provide additional sensitivity to these small features as the scattering from the domain walls contributes to the resonance peaks, while the magnetic orientation and moment creates the asymmetry in the scattering between two incoming polarizations. X-PEEM measurements indicate a wall width that is about a factor 2 wider, but include the full extension of the domain wall tail [15,41]. The combination of a narrow core and wide extended tail is not resolved in the neutron experiment, which approximates the magnetic potential as top-hat functions with sharp edges. Therefore, the discrepancy may be explained by the different description of the wall shape. A more complicated line shape of the domain boundaries is currently not justified by the data, but may include a power law function to describe a roughness or slow rotation in an extended tail. 

Previous measurements on imprinted magnetic stripe patterns did not detail the observation of domain walls or resonance features [32,33]. This can be explained, in part, by the different magnetic orientation. For the side-by-side orientation, a bi-polar stray field originating from the magnetic charges in the domain walls has been determined [12,14]. This bipolar nature can lead to a diminished contrast, as the sense of rotation with respect to the external field is inverted depending on the side of the domain wall, and therefore could remain below the detection limit in previous experiments. For the present hth configuration, the sense of rotation is the same for either side of the domain wall, creating a larger contrast towards the center. For the transition between two hth domains, two directions are energetically degenerate. However, within the IBMP pattern, we only observe a rotation into one defined direction. This direction is likely defined by the small offset in exchange bias direction of the two domains during preparation, but could also be caused by the field history of the sample. 

In contrast to previous studies, we do not observe any indication of ripple domain formation, which would lead to pronounced diffuse OSS [32,64]. In topographically patterned magnetic stripes, these ripples were attributed to an irregularity of the anisotropy in wide stripes due to the size of the pattern [64]. The continuous nature of the IBMP sample circumvents this limitation, but creates competing in-plane direct exchange and interfacial AF/FM exchange interactions. In experiments on IBMP samples with side-by-side magnetization vectors, ripple domains were observed during the magnetization reversal of the stripes [32,33]. Based on the behavior of these ripples with external field, it was concluded that the magnetization reversal takes place via domain nucleation and propagation, rather than coherent rotation of the stripe magnetization. In the present case, no ripple domains were observed, which could therefore indicate a different reversal mechanism of the sample. The single image of the domain state may not be fully conclusive to support this statement, but a field dependent study of the scattering has been recorded suggesting a continuous rotation of magnetization vectors and absence of diffuse OSS due the formation of significant ripple domains [64]. 

## 5. Conclusions and Outlook

Using PNR and polarized OSS a quantitative description of the structural and magnetic arrangement of an exchange bias system with engineered domain texture is obtained. The technique resolves the layers as a function of depth in the sample and further quantifies magnetization vector directions and lengths in different parts of the structure, laterally and in depth. We determine an in-plane structure of two magnetic domains tilted by 89° ± 3° and 82° ± 3° with respect to the external magnetic field and long axis of the stripe. The laterally averaged magnetization is tilted by 5.7° ± 1.5° against the external field. The domains show a different width of (5.3 ± 0.1) μm and (3.7 ± 0.1) μm, respectively, and are separated by domain walls of (0.6 ± 0.1) μm width whose magnetization direction uniformly falls along the stripe axis. We do not detect significant changes in the chemical layer structure or saturation magnetic moment due to the light-ion bombardment. The stripe pattern shows a defined sense of rotation of individual magnetizations, which is related to a misalignment in the preparation process or due to the field history of the sample. The latter will be tested against field dependent measurements in future studies. Together with measurements of stripes with different width, further information about the magnetic domain wall and the magnetic domain stability will be obtained. Especially, if the measured domain wall size becomes comparable to the domain width, one expects a larger degree of disorder across the domain, which will reflect in the scattering pattern and can be quantified as s function of field.

The quantitative information in absolute units provided by the PNR and OSS highlights the details that can be obtained by PNR and OSS from three dimensional textured systems on nano- to micrometer level. Values of film thickness, roughness, density and magnetization are obtained in absolute units without the need of prior calibration. PNR provides depth resolved profiles on sub-nanometer level without signal damping for buried interfaces. Using OSS, lateral textures showing a variation in chemical or magnetic properties on micrometer length scales can be resolved with nanometer resolution. The results are complementary to volume averaged and microscopy techniques, such as X-PEEM and MFM, which only provide near surface information or rely on image contrast and calibration to provide absolute units. The combination of absolute units in length and magnetization provided by PNR and OSS provides valuable additional information on the magnetization distribution and layer structure in depth and laterally that cannot be obtained from the combination of VSM, X-PEEM and MFM alone.

The technique is applicable to a wide range of material classes (See for example References [31,34,35,36,37,38,39] and references therein). For example, magnetization vector distributions along the surface normal or along the sample plane can be obtained from thin-film systems comprising exchange coupled hard-soft magnetic hybrid materials and phase separation [36,65,66,67], exchange coupled magnetic multilayers [38,68] and non-magnetic thin film systems [31,37,69]. PNR and OSS can further be used to detect correlations among magnetic domains as a function of depth and laterally in nanoparticle systems or emerging material classes, such as topological insulator [70,71] and multiferroic nanostructures [72].

## Figures and Tables

**Table 1:** Structural and magnetic parameters determined from fitting the specular data. Values in parenthesis are nominal or literature values determined from tabulated densities and scattering length of the materials [52].

Material	Thickness (nm)	Roughness (nm ± 0.2 nm)	N-SLD (10^−6^Å^−2^)	iN-SLD (10^-9^Å^−2^)	M-SLD (10^−6^Å^−2^)
TaOx	3.1 ± 0.3 (-)	1.7	5.54 ± 0.7 (4.85)	3.3 ± 20 (3.2)	-
Ta	8.3 ± 0.2 (10)	0.5	3.52 ± 0.4 (3.83)	3.3 ± 20 (3.2)	-
Co_70_Fe_30_	6.1 ± 0.1 (10)	1.3	4.17 ± 0.1 (4.00)	3.7 ± 10 (6.1)	4.88 ± 0.1
IrMn_3_	29.6 ± 0.1 (30)	1.5	−0.74 ± 0.05 (−0.96)	18.2 ± 5 (17.2)	0.12 ± 0.1
Cu	3.7 ± 0.3 (5)	1.2	5.57 ± 0.5 (6.53)	0.7 ± 10 (0.9)	-
SiO_2_	1.0 ± 0.2 (-)	1.4	3.66 ± 0.2 (3.66)	0.012 ± 15 (0.01)	-
Si	Substrate	0.4	2.1 ± 0.2 (2.07)	0.023 ± 1 (0.024)	-
